# The Influence of the Nutritional and Mineral Composition of Vegetable Protein Concentrates on Their Functional Properties

**DOI:** 10.3390/foods14030509

**Published:** 2025-02-05

**Authors:** Rocío López-Calabozo, Iván Martínez-Martín, Marta Rodríguez-Fernández, Yamina Absi, Ana María Vivar-Quintana, Isabel Revilla

**Affiliations:** 1 Food Technology, Polytechnic High School of Zamora, Universidad de Salamanca, Avenida Requejo 33, 49022 Zamora, Spain; rociolc@usal.es (R.L.-C.); ivanm@usal.es (I.M.-M.); martarf98@usal.es (M.R.-F.); absiyamina@usal.es (Y.A.); irevilla@usal.es (I.R.); 2 Independent Researcher, 49029 Zamora, Spain

**Keywords:** soybean, rice, pea, protein concentrate, techno-functional, biplot analysis, mineral content

## Abstract

Vegetable proteins derived from legumes, cereals or pseudocereals have increased in popularity in recent years, becoming very interesting for the food industry. In addition to their nutritional interest, these products have techno-functional properties that allow them to be used in the production of a wide variety of foods. This research has studied the nutritional and mineral composition of 12 samples of rice, pea and soy concentrates. The objective was to investigate the influence of this nutritional composition, mainly mineral components, on the techno-functional properties (water- and oil-binding capacity, swelling, emulsifying, gelling and foaming capacities) of these concentrates. For this purpose, a Pearson correlation matrix and a GH biplot method were applied. The results showed that there is a correlation between mineral content and functional properties. Mg, K and Ca were positively correlated with protein solubility index, oil absorption capacity and swelling capacity. Na and P contents were positively related to water absorption capacity and emulsifying capacity. Gelling capacity was positively correlated with Mg contents and negatively correlated with Cu and Fe contents. The preliminary results reported in this study highlight the necessity to further assess the influence of non-protein components on the techno-functionality of protein concentrates.

## 1. Introduction

The increasing demand for sustainable and plant-based protein options has propelled vegetable proteins into the spotlight thanks to their exceptional nutritional composition, functional properties and potential health advantages [1]. Vegetable Protein Concentrates (VPCs) are products derived from plant sources that have been processed to increase their protein content. The protein content of VPCs can range from 40% to 90% by dry weight; these percentages vary depending on the processing methods employed, as well as the plant source [2]. These products generally have a favourable proximate composition, characterised not only by high protein content, but also by low levels of fat and the presence of bioactive compounds, which occur naturally in plant sources and are associated with potential health benefits [3]. In addition, they exhibit functional properties such as water- and oil-holding capacity, swelling capacity, foam formation and/or gelling; these functional properties allow the application of vegetable proteins for the development of new products, replacing animal proteins totally or partially [4].

Among the plants most commonly used to obtain VPCs are legumes, cereals and pseudocereals [5]. One of the best-known and most widely applied sources of vegetable protein in the food industry is soybean. Its composition shows elevated levels of cysteine, methionine and tryptophan and a high content of minerals such as zinc, iron and copper [6]. From a nutritional point of view, the partial substitution of wheat by soy increases the protein content and improves the nutritional value of foods [7]. This replacement enriches the amino acid profile of the final products, particularly by covering lysine and tryptophan deficiencies in cereals [8]. Soybean protein applications include bakery products, snacks, bars and beverages [9] and their incorporation into corn tortillas [8]. Other applications of soy proteins are the production of miso, cheese, tofu and meat-like vegetarian products [10].

In addition, VPCs from legumes are gaining popularity in the food industry due to their richness in essential amino acids and their competitive price [11]. In recent years, there has been increasing interest in pea proteins due to their sustainability, low allergenicity and balanced amino acid profile [12]. These proteins are being widely used in the food industry to manufacture protein bars, gluten-free confectionery and bakery products, and pasta, and even as a substitute for egg proteins in mayonnaise [3].

Some cereals such as rice are other important vegetal sources of protein. Rice protein shows high nutritional value and hypoallergenic properties and has been described as higher quality than wheat and maize [13]. Industrial applications of rice protein concentrates include their use in bakery and biscuit products, the production of alternative beverages for children with cow’s milk allergies, the development of supplements for athletes [14], and the production of products for infants and the elderly [15].

The composition and functional properties of VPCs are influenced by the raw material used [16], but also by the production process employed [17]. This results in the fact that different commercial brands, even obtained from the same raw material, can differ significantly [18]. For this reason, the industries must face the challenge of optimising their production processes whenever they change suppliers. In this context, the present work tries to evaluate the relationships between the physicochemical composition of protein concentrates and their techno-functional behaviour. For this purpose, linear correlations, through a correlation matrix, as well as the effectiveness of multivariate GH biplot techniques were tested as a tool to evaluate the links between composition and functionality.

## 2. Materials and Methods

### 2.1. Materials

A total of 12 samples corresponding to six commercial brands of protein concentrate, two soybean (*Glycina maxima*), two pea (*Pisum sativum*) and two rice (*Oryza sativa*), were analysed. Samples were purchased through online shops. Each commercial brand was purchased twice, with 6 months in between, in order to account for production variability. To avoid conflicts of interest, the brands have not been identified; instead, they have been named Brand 1 and Brand 2.

### 2.2. Proximate Composition

To determine the nutritional composition of the samples, the methods described by Absi et al. [18] were applied. The parameters analysed were moisture, fat, protein, fibre, starch, ash and carbohydrates. The Soxhlet method was used for fat determination using petroleum ether. Protein content (N × 6.25) was determined by the Kjeldahl method. Although N conversion factors between 5.66 and 5.79 have been recommended for soy protein [19], the N of 6.25 is still widely used in the nutrition labelling regulations of Codex Alimentarius and other governmental regulatory agencies in different countries. In addition, the 6.25 N conversion factor for soy protein analysed by Kjeldahl methods is recognised by technical associations, such as the American Oil Chemists Society (AOCS), AOAC International (AOAC), the AACC International (AACC) and the International Organization for Standardization (ISO) [20].

Ash was determined by incineration at 550 ± 10 °C, and dietary fibre was analysed using ANKOM equipment (ANKOM Technology, Macedon, NY, USA). The enzymatic method described in AOAC 996.11, hydrolysing starch to maltodextrins with thermostable α-amylase at 95 °C, was used for the determination of starch content. The gravimetric method AOAC 925.10, drying at 105 °C in an air oven, was used to analyse the moisture content [21]. Total carbohydrates were calculated by difference, and the amount of energy provided was calculated considering that protein and carbohydrates contribute 4 kcal/g, and fat 9 kcal/g. All determinations were carried out in triplicate. Results were expressed in g/100 g dry weight (dw).

### 2.3. Mineral Analysis

Mineral composition was analysed by inductively coupled plasma mass spectrometry (ICP-MS). A microwave pre-digestion with HNO_3_ was performed using 0.2 g of sample. The analysis was carried out with an Agilent 7800 ICP mass spectrometer (Santa Clara, CA, USA) according to the method previously described by Absi et al. [18]. Certified standard solutions (1 g/L) were used for quantification and results were expressed in ppm or ppb depending on the mineral.

### 2.4. Physicochemical Parameters

The water activity (aw) of the samples was determined in triplicate using Novasina AW Sprint TH500 equipment (Lachen, Switzerland). Colour determination was carried out using a MiniScan XE Plus (Hunterlab, Reston, VA, USA), with a D65 illuminant and a 10° standard observer. The colour parameters L*, a* and b* were recorded. The analysis was performed in nine replicates for each sample.

### 2.5. Techno-Functional Properties

#### 2.5.1. Water-Holding Capacity, Oil-Holding Capacity and Water Solubility Index

The methods described by Rodríguez-Miranda et al. [22] were used. For water-holding capacity (WHC) determination, the sample (0.5 g) was mixed with 10 mL of distilled water and vortexed for 30 s. After 24 h of standing at room temperature, the sample was centrifuged for 20 min at 3000 rpm in a Sigma 4K 15C centrifuge (Darmstadt, Germany). The supernatant was discarded, and the weight of the moist sample was determined. The analysis was performed in triplicate and the results were expressed as g/g of sample, calculated following Equation (1).

(1) $$WHC=\frac{W_{2}-W_{1}}{W_{0}}$$

where *W*_0_ is the weight of the dry sample (in g), *W*_1_ is the weight of the tube plus the dry sample (in g), and *W*_2_ is the weight of the tube plus the sediment (in g).

A similar procedure was used for the determination of the oil-holding capacity (OHC), where 0.4 g of sample was mixed with 5 mL of olive oil and vortexed. After storage and centrifugation, as described for WHC, the sample weight was determined after removal of the supernatant. The results were expressed in g/g and the analysis was performed in triplicate, and calculated following Equation (2):

(2) $$OHC=\frac{W_{2}-W_{1}}{W_{0}}$$

where *W*_0_ is the weight of the dry sample (in g), *W*_1_ is the weight of the tube plus the dry sample (in g), and *W*_2_ is the weight of the tube plus the sediment (in g).

The water solubility index (WSI) was determined with a portable refractometer ZUZI 300 (Barcelona, Spain) in the supernatant obtained in the WHC determination. The analysis was performed in triplicate and the results were expressed in ºBrix.

#### 2.5.2. Swelling Capacity

The swelling capacity (SC) was determined following the method described by Akpata and Miachi [23] with some modifications. The sample (0.5 g) was mixed with distilled water (5 mL) and vortexed for 1 min. After storage for 24 h at room temperature, the final volume of the sample was determined. The analysis was carried out in triplicate. The results were expressed as volume increase per gram of sample.

#### 2.5.3. Foaming Capacity

The method proposed by Chau et al. [24] was used to determine the foaming capacity (FC) with some modifications. For this purpose, an ULTRA-TURRAX T 25 basic homogeniser (IKA-WERKE, Staufen, Germany) was used, and the sample (5 g) was stirred (19,000 rpm, 2 min) together with 100 mL of distilled water. The stability of the foam formed (FS) was determined from the foam remaining after 5 min at room temperature. In both FC and FS, the height of the foam was determined, and the result was expressed as a percentage. All analyses were carried out in triplicate.

#### 2.5.4. Gel Formation

Gel formation was evaluated from the hardness of the gel produced by the sample following the method described by Tomé et al. [25]. The gel was formed by mixing the sample (6 g) with 3 g of sodium chloride (Scharlab SL, Barcelona, Spain) and distilled water (30 mL). The samples were subjected to heating at 90 °C for 30 min and subsequently refrigerated for 24 h prior to texture determination. A penetration test with a 10 mm probe was performed in a Texture Analyser TX-T2iplus (Stable Micro Systems, Surrey, UK) with a 5 kg load cell at 20 °C. A penetration distance of 8 mm and a crosshead speed of 1 mm/s were used. The mean maximum force from three replicates was recorded.

#### 2.5.5. Emulsifying Activity and Its Stability

The emulsifying activity (EC) and stability (ES) were determined using the methodology described by Bora [26]. For the determination of the EC, the sample (2.25 g) was dissolved in 150 mL of distilled water. The mixture was then homogenised with 10 mL of sunflower oil (13,000 rpm, 1 min) using an Ultraturrax (ULTRA-TURRAX T 25 basic, IKA-WERKE, Staufen, Germany). The resulting emulsions were centrifuged (Sigma-Aldrich 4K15C, Darmstadt, Germany) at 1500 rpm for 5 min at a temperature of 15 °C. The ES was determined after heating the sample at 80 °C for 30 min and then subjecting them to centrifugation under the same conditions. The EC and ES were determined in triplicate, expressed as a percentage and calculated following Equations (3) and (4).

(3) $$EC\;(\%)=\frac{Height\;of\;emulsified\;layer}{Height\;of\;the\;contents\;of\;the\;tube}\times 100$$

(4) $$ES(\%)=\frac{Height\;of\;remaining\;emulsion\;layer}{Height\;of\;original\;emulsified\;layer}\times 100$$

### 2.6. Statistical Analysis

Variations between samples were evaluated using one-way analysis of variance, followed by Tukey’s significant difference post hoc. The data were analysed using IBM SPSS Statistics (version 27). The correlations between the different physicochemical parameters and the techno-functional properties of the different VPCs analysed were studied using a Pearson two-tailed significance correlation. Finally, the multi-parametric GH biplot method was used to describe the relationships between protein concentrates of different raw materials and the physicochemical and techno-functional variables analysed. GH biplot is a method of graphical representation of multivariate data in which the axes composing the reference system are the principal components of the indicator space [27]. A free-application R package MULTBIPLOT programme package (MULTivariate analysis using BIPLOT) developed by Vicente-Villardón [28] was used for statistical analysis.

## 3. Results

### 3.1. Proximate Composition

The proximate composition and content of rice, pea and soy protein concentrates are shown in Table 1. The moisture content of all the samples was low, ranging from 4.3% to 7.2%, with pea concentrates showing the highest moisture content, regardless of the commercial brand. In all the samples, protein accounted for between 84 and 89%/dw of their composition, with soybean concentrates having the highest contents and pea concentrates the lowest. The fat content varied between 9.3% (pea) and 2.6% (soybean); these values were lower in all cases than the fat content of the raw materials from which they were derived. Carbohydrates were present in low concentrations; however, one of the commercial brands of rice had a content of 7.36%, with this sample presenting the lowest ash content. The starch concentrations found ranged from 0.9% to 2.0%, while the fibre and sugar contents of all samples were below the detection limits. Soybean concentrates had the lowest energy content due to their low fat content.

Regarding moisture content, values obtained are within the ranges described for VPCs in the bibliography varying between 2.25% [14,17] and 17.03% [29]. Moisture contents are high for pea concentrates and lower for soybean and rice. Zhao et al. [30] found higher moisture content in pea concentrates, followed by soybean, and significantly lower in the case of rice concentrates. Also, de Paiva Gouvêa et al. [31] described higher moisture content in pea concentrates than in soybean ones. The extraction process of protein concentrates seems to be the main factor influencing the moisture content of protein concentrates [32]. According to Mondor et al. [33], protein concentrates have a protein content between 65 and 90% *w*/*w* on a dry basis, while for contents higher than 90% *w*/*w,* the denomination used is protein isolates. Based on these classifications, the products analysed in this research can be considered protein concentrates, with a high protein content, above 84% *w*/*w* in all of them. However, a large variability is found in protein concentration in VPCs in the literature. Thus, for rice protein, concentrations between 67% [29,34] and 83.57% [17] have been described, while for pea protein, the described range is much wider, being between 48.5% and 82.0% [25,35]. In the case of soybean, higher amounts of protein have been described: Zhao et al. [36] reported a protein content of 86.36%, while Foh et al. [37] found amounts of 88.66%.

Fat contents also show great variability; values between 0.36% and 2.0% have been described in VPCs [17,25,35,37]. The fat content of these types of compounds is related to the defatting process to which they are subjected; the results obtained in the samples analysed seem to indicate partial defatting as they still retain high fat contents, particularly in the case of rice concentrate.

Ash content was found to be similar to the values described by Zhao and Boatright [17] (0.96% to 2.31%), Amagliani et al. [14] (2.35 to 9.55%), Reda et al. [34] (2.5%) and Guroy et al. [29] 4.78%. Similarly, a wide range of fibre (0.72 and 2.02%), starch (0.39 to 6.50%) and carbohydrate (9.03 to 16.2%) contents have been described for this type of product [14,29]. For all the protein concentrates analysed, there are significant differences (*p* < 0.05) between the brands of each sample. The biggest differences were found between the soybean and rice protein brands in relation to moisture and carbohydrate content. The variability found in the parameters studied is related to different raw material factors such as genetic background, annual climatic conditions and crop location, as well as the interaction between these [38]. Besides this, factors related to the process of obtaining the concentrates also have a great influence on their final composition [39].

### 3.2. Mineral Content

Fourteen minerals, including macroelements (sodium, magnesium, phosphorus, potassium, and calcium), microelements (manganese, iron, copper, zinc, chromium, selenium, nickel), and trace toxic elements (cadmium, and lead), were analysed in rice, pea and soybean protein samples (Table 2). The highest concentration of minerals in all the analysed VPCs was found for the macroelements Na, P, and Ca. Pea and soybean concentrates show Na as the most abundant mineral, followed by P and Ca. In the case of rice concentrates, P is the most abundant mineral. All these minerals showed significant differences between the two commercial brands.

In relation to micronutrients, Fe is the one found in the highest concentration in the samples analysed, followed by Zn. The Fe concentrations were higher than 80 ppm in all concentrates, highlighting the high concentrations found in pea concentrates. For all concentrates, significant differences were observed between both commercial brands, being particularly striking in the case of rice concentrates where the Fe content was double in one of the commercial brands. Regarding other micronutrient contents, the concentrations of Se and Ni stand out, with a high concentration of Ni present in the soybean concentrates. For these minerals, the differences found between commercial brands are greater than those found for macronutrients, as well as the differences between batches of the same commercial brand. Cd was not found in any of the samples analysed and Pb was only found in one of the samples corresponding to a commercial brand of rice concentrates.

The legume concentrates (soybean and pea) showed the highest concentration of macroelements, which contributed to their higher ash values (Table 1). In relation to microelements, pea concentrates presented the highest concentration; only the Ni content was higher in soybean concentrates. The greatest differences between commercial brands were found in the micronutrient composition of rice concentrates, mainly due to variations in Ni and Cr content.

Similar mineral contents have been described for soy protein concentrates, with K and P being the most abundant minerals [40]. Karr-Lilienthal et al. [41] found P to be the major mineral in soybean concentrates with high concentrations of K and Ca. Also, in rice concentrates, P has been described as the major mineral with important concentrations of K and Ca [42]. For pea concentrates, P, K and Mn have been described as the major minerals, with important Ca contents as well [43]. Large differences have been described between concentrates in the Na and K content, which could be related to the incorporation of NaOH or KOH to adjust the pH during the enzymatic hydrolysis reaction in the production of protein concentrates [44]. Regarding micronutrients, previous studies agree with our results, finding that iron is the major micronutrient in rice [17] and soybean [45] concentrates.

Several factors could explain the differences found in the mineral composition of VPCs between commercial brands and/or production batches. Firstly, the cultivar used to obtain the concentrate will influence its mineral content [46]. On the other hand, many minerals are incorporated into grains depending on the available mineral concentration found in the soil, so the mineral content of the soil and/or its availability in plant tissues also influence the mineral content of concentrates [47]. Karr-Lilienthal et al. [41] observed, in soybean concentrates, a great difference in the contents of micronutrients and trace elements depending on the country of origin of the concentrate, attributing these differences to the mineral composition of the soils. In addition, the production process of the protein concentrate could influence the final concentration of minerals [45]. Thus, an effect of Fe and Mn concentration attributed to the process of obtaining rice concentrates has been described [17], and differences in the contents of Cu, Co, Mn and Ni due to the process of obtaining pea concentrates have also been reported [48]. In addition, processes such as air sorting have been shown to have an important influence on the mineral concentrations of protein concentrates [44,49].

Regarding the presence of Pb in rice concentrates, there are no previous studies that have analysed the presence of Pb in these products. However, the presence of this toxic mineral has been described in rice grown in different countries [50] and in rice flour [51]. Despite protein extraction treatments, Pb can be present in this type of product as highlighted by Irshad et al. [52], where the presence of Pb was described for a wide variety of protein supplements used for muscle growth. Pb would reach these products through plants contaminated by irrigation water, fertilisers and pesticides but also through contamination originating from industrial activity and/or busy roads [53].

### 3.3. Techno-Functional Properties

The behaviour of protein concentrates during the preparation, processing, storage and consumption of the foods in which they are incorporated is determined by their functionality. This functionality is influenced by the raw material the concentrate comes from, as well as the protein concentration and the technological extraction process itself [54]. In this study, surface properties such as protein solubility, foaming capacity, emulsifying capacity and liquid-binding capacity were analysed. In relation to the hydrodynamic properties, the gelling capacity was determined. The results obtained are shown in Table 3.

The rice concentrates show the lowest values for water-holding capacity (WHC), oil-holding capacity (OHC) and water solubility index (WSI). Soybean and pea concentrates show similar values for WHC and OHC; however, WSI is significantly higher for soybean concentrates. WHC and OHC properties measure the amount of water or oil that can be retained per unit mass, while WSI determines the soluble solids, measured as °Brix, that remain in the aqueous phase. The soluble solids measured in the aqueous phase are mainly protein, but other hydrophilic compounds present would also be measured, so this parameter is closely related to the chemical composition of the concentrate [44]. In the case of soybean, the higher WSI could be related to its higher ash content, highlighting its high concentrations of Na, K, Mg, K and Zn (Table 2). On the other hand, the protein content could affect the solubility of the protein product, with a higher protein content being observed in soy concentrates (Table 1). Differences between commercial brands can also be observed for these properties in the case of soybean concentrates. These differences may be due to the fact that protein solubility is highly related to the extraction process [55], due to the influence of the process on the relationship between the number of polar and apolar groups and their arrangement along the molecule [56]. Thus, treatments that result in greater exposure of hydrophilic regions of proteins that interact with water result in concentrates with higher solubility [57].

Swelling capacity (SC) refers to the amount of space taken up by a specific quantity of hydrated protein [58]. The results obtained (Table 3) show that soybean concentrates have the highest SC followed by pea, with the values for rice concentrates being significantly lower. However, for the foaming capacity (FC), soybean concentrates have the lowest capacity. The rice concentrate, in addition to having a higher FC, also has a higher foam stability (FS). The FC describes the amount of interfacial area that proteins are able to stabilise per unit weight [14]. Foam formation (FC) has been related to the amount of protein present in the concentrates, so FC increases with higher protein content [59]. However, although the soybean concentrates analysed in this study have a higher protein content, their FC is lower than that of the pea concentrates, although they have a higher stability of the foam formed (FS) (Table 3). De Angelis et al. [54] pointed out that the FC could be affected by the presence of non-protein components in the concentrates. Thus, the lower foaming capacity and stability of soybean may be related to its nutritional composition (Table 2) because the presence of high lipid content seems to inhibit the ability of protein to form and stabilise foam, and on the contrary, starch has a positive impact on foaming properties due to its ability to stabilise foam [60]. On the other hand, Adebiyi and Aluko [61] and Chavan et al. [62] pointed out that the foaming capacity seems to be only related to the soluble proteins in the aqueous phase since these proteins, thanks to their flexibility, would form an interfacial membrane between the air bubbles and the aqueous phase. The emulsifying capacity (EC) shows no differences between concentrates, and for emulsion stability (ES), rice concentrates seem to have a higher stability. The EC is defined as the volume of the emulsified layer after centrifugation of the emulsion, while emulsion stability (ES) is a measure of the emulsion stability over a certain time [54]. Non-protein components, such as fibre, have been described as factors with a positive effect on ES [63]. All these properties are strongly influenced by pH; in our case, the determination of these properties was carried out at the native pH of the concentrates, which had a pH value of pH ≈7. Adebowale et al. [59] showed that properties such as foam formation and emulsification are affected by protein solubility. From our results, although soy concentrates have a higher WSI, their FC and EC are lower than those found in pea concentrates. Regarding gelation, statistically significant differences in the hardness of the gels formed at pH 5 can be observed, with soybean concentrates forming the hardest gels followed by pea. The gels of the rice concentrates were much less hard, showing differences between the two commercial brands. Gelation involves the transition from a liquid to a solid state, characterised by a three-dimensional matrix in which the liquid phase is retained. Gel formation requires proteins to denature and unfold to form a network capable of retaining water molecules [54]. The gelation capacity is closely related to the protein content, but other components present can influence this property, so the starch and lipid content negatively affect gelation [60]. Regarding starch, no significant differences were observed between the concentrates (Table 1); however, soybean concentrates showed the lowest fat content, which could explain the higher hardness of the gel formed. The EC, FC and gelling properties are influenced by the water solubility of the proteins [64]. This solubility is in turn influenced by the botanical origin, by the presence of other compounds such as polyphenols, and by the extraction method employed [65].

In addition to the functional properties, the parameters water activity (aw) and colour (L, a* and b*) were also determined (Table 3). The aw was found to be between 0.3 and 0.4 for all concentrates, with the exception of soy Brand 2, which showed values of 0.134. Significant differences were observed for this parameter depending on the raw material, but also between the commercial brands. Regarding colour, all the concentrates showed high L values (>70) and positive a* and b* values, indicating that their colour was light and tended to red and yellow. The highest values for these two parameters were observed for the pea concentrates. Significant differences in the colour parameters both between raw materials and between commercial brands were observed.

As pointed out by De Angelis et al. [54], the comparison of properties in different studies is very complicated as it depends on the methodologies used for their determination. However, some comparisons can be carried out; for instance, for the rice concentrates, similar results have been described by Amagliani et al. [14] for OHC and EC properties, stating that the low surface hydrophobicity of rice concentrates would be the reason for these results as it would cause weak interactions between proteins and oil [66]. Regarding the foam formation values, the results obtained in our work are lower than the FC (116%) and FS (98%) values described by Zhao et al. [36]. For pulses, the functional properties of concentrates vary depending on the legume from which they are derived [67]; thus, peas have been shown to have a higher FC than other legumes [65]. Soybean has been described as having a good EC and a good FC [9]. However, our results show that there were no significant differences in EC with respect to other concentrates, presenting a low FC value. In relation to pea, good EC, FC, WHC and OHC have been described [68], with a weaker gel texture compared to soy proteins [45], which would be consistent with the results found in our samples.

As already noted, the physicochemical composition of protein concentrates significantly influences their techno-functional properties; factors such as protein purity or the presence of compounds other than the protein itself influence these properties. The source of the protein (animal or plant) and its intrinsic characteristics also play a crucial role. Legume proteins have a low proportion of hydrophilic amino acids with a compact structure that limits their solubility and emulsifying capacity [69]. In contrast, animal proteins, such as whey, exhibit good gelation due to their globular structure and higher sulphur content [70]. The higher soluble protein content improves the ability to form and stabilise emulsions and foams, while the presence of residual polysaccharides can affect the viscosity and gelling properties of the concentrates [71]. The stabilisation of emulsions is enhanced by the presence of non-protein components such as dietary fibre [63]. Foaming capacity is positively influenced by the presence of high contents of albumin-like protein [72] and starch [73], while the presence of lipids can negatively influence this property [60]. The mineral content, in combination with low-molecular-weight peptides and amino acids, has been shown to have an impact on the thermomechanical behaviour in the extrusion process [73]. Additionally, the chemical or physical modifications during the production processes also affect the techno-functional behaviour. As such, protein solubility is negatively affected by processing conditions such as heating or an acidic or alkaline environment, while the ability to bind water is enhanced when using wet-extraction technologies [74]. Hydrodynamic properties are also affected by the production process, with the strongest gels being obtained when using wet-extraction processes [75]. In addition, it has been found that enzymatic hydrolysis or conjugation with polysaccharides can improve some techno-functional properties of protein concentrates [76,77].

### 3.4. Correlations Between Physicochemical and Functional Parameters

**Figure 1:**
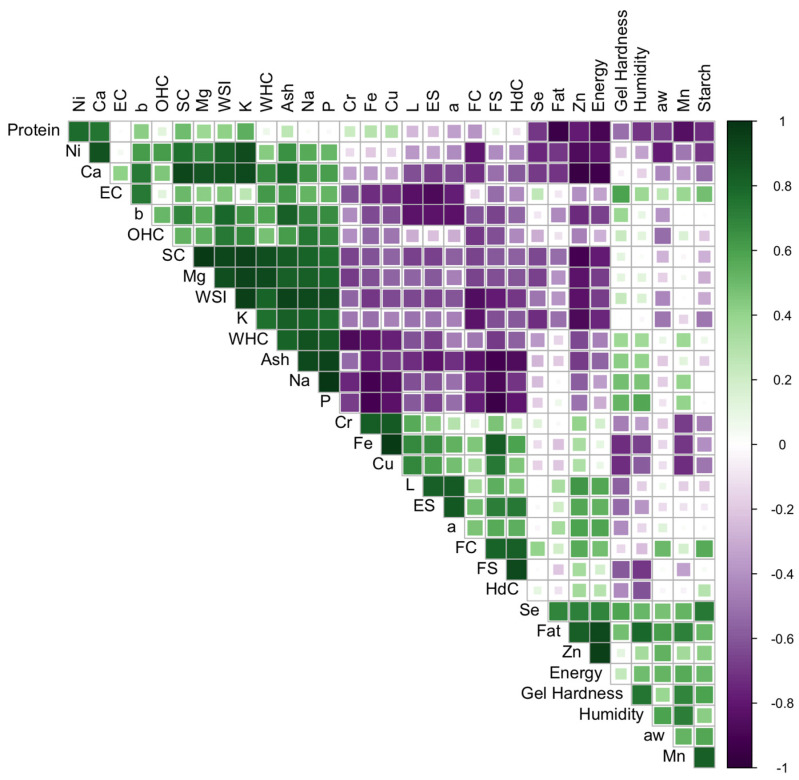
Heat map of Pearson correlation coefficients of protein concentrates between the physical–chemical and mineral parameters and the techno-functional properties analysed.

A pair-wise correlation analysis was conducted to investigate the interaction between the physicochemical parameters and techno-functional properties of soybean, rice and pea protein concentrates (Figure 1). Regarding the correlations between the mineral compounds, significant strong correlations were observed between several minerals, being positive for K with Mg (0.93) and Ca (0.91), and for Fe with Cu (0.97), and negative in the cases of Fe with Na (−0.90) and P (−0.90) and Zn with Ca (−0.95). Significant correlations between 0.8 and 0.9 were also found, both positive and negative, mainly for Ca and Mg with several macro and microelements (Figure 1). Correlations between different minerals have already been highlighted in some concentrates; in rice protein concentrates, a positive correlation between iron and magnesium content has been described [17].

Regarding the correlations between the mineral composition and the techno-functional properties, it can be observed that the Na and Mg contents are significantly correlated, in a positive way, with almost all the functional properties of the concentrates, except with the emulsifying capacity and gelling. In the case of Mg, there is also no significant correlation with OHC. The highest correlations are observed for WHC with Mg (0.91); SC with Mg (0.97), K (0.94) and Ca (0.92); and WSI with Na (0.91) and K (0.95). Emulsifying capacity and gelling capacity only correlate with Fe and Cu contents (−0.72 for both minerals). Significant negative correlations were also observed between Zn content and SC (−0.90) and P content and FS (−0.94). As noted previously, the techno-functional properties are influenced by the composition of protein concentrates; the minerals belong to the group of so-called antitechnological factors (ATFs), due to their interaction with proteins [57]. According to our results, emulsifying capacity, gelation, FC and SC showed a negative correlation with some of the minerals analysed. However, the correlation between the macroelements in the concentrates and the techno-functional properties was positive for most of these properties, with the SC and WSI properties standing out.

In relation to the correlations between the different techno-functional properties, there are significant positive correlations between the properties WSI and WHC, and between SC and OHC, and a negative correlation with FC. The WHC and SC are properties that are closely related to protein solubility, so it is reasonable that they are correlated with WSI. The colour parameters correlate significantly with the EC and ES properties and the b parameter also with WSI (0.79). In addition, the FS property is positively related to FC and ES. The good correlation between these properties may be due to the fact that the formation and stability of foams and emulsions are closely related to hydrophobic interactions, which are favoured by the amount of hydrophobic amino acids in the concentrate; this would also explain the negative correlation with WSI since these amino acids reduce the interactions with water, interfering in its solubility [78]. A relationship between WSI and other properties such as EC, FC and gelation has been previously reported [79].

In order to evaluate linear relationships or links between variables different from those provided by the usual studies of correlation coefficients, it was decided to apply the multivariate technique GH biplot. Biplots are graphical techniques that allow the visual identification of real associations between variables which have proven to be suitable for the study of compositional variables in food [80]. This method allows us to project the original data onto a lower-dimensional subspace, so that most of the variability can be captured. The main difference between biplot methods and a traditional PCA is that it allows us to represent both variables and samples. Among the biplot methods, the GH biplot is characterised by the fact that it achieves a high-quality representation of the variables [81].

**Figure 2:**
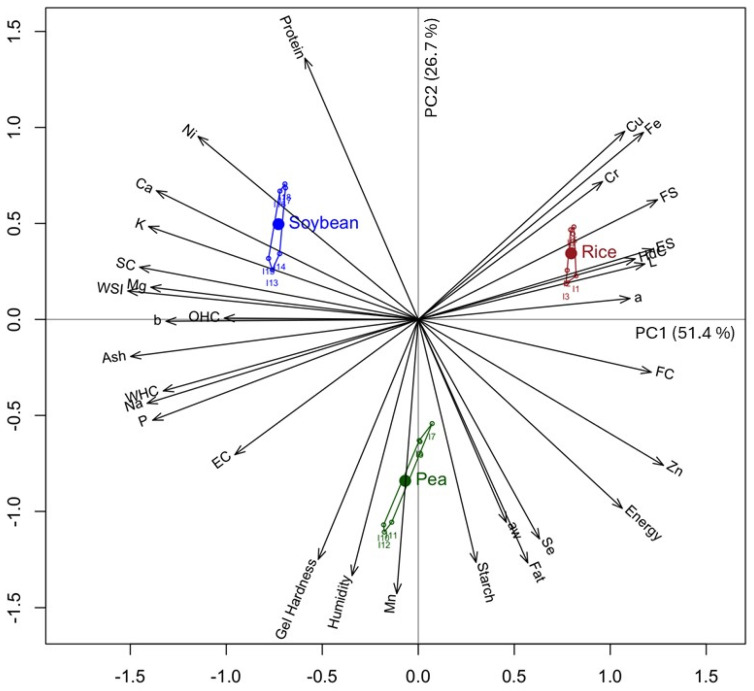
GH biplot based on the principal component (PC1 51.4–PC2 26.7%) analysis for nutritional composition parameters and physicochemical and techno-functional properties of the protein concentrates analysed.

Figure 2 shows the representation of the factorial plane (PC1, PC2) resulting from the GH biplot diagram, which explains 78.1% of the variance. The physicochemical and techno-functional variables are represented as vectors and the protein concentrates as points. The length of the vector is related to the standard deviation, and the cosine of the angle between two vectors indicates the correlation between the variables.

A clear segregation between the protein concentrates analysed as a function of the raw material is shown in Figure 2. The principal component PC1, which explains 51.4% of the variance, allows us to differentiate between rice concentrates with positive values on this axis and soybean concentrates with negative values. The greatest contribution to this axis corresponds to techno-functional properties and minerals. The functional properties are mainly those related to protein solubility such as SC, WSI and WHC, and all minerals have a great weight on this axis except Mn and Se. PC2, which explains 26.7% of the variance, allows us to differentiate the pea concentrates with negative values from the soybean and rice concentrates with positive values on this axis. The greatest contribution to this axis corresponds to nutritional composition parameters such as protein, starch and fat; for minerals, Mn has a great contribution, and regarding functional properties, gelling stands out. Considering the vectors that are closer to the concentrates, it should be noted that in terms of minerals, rice concentrates are located near the Cu and Fe vectors, soybean concentrates are located near the Ca, K, Ni and Mg vectors, and pea concentrates are located near the Mn vectors. Regarding the techno-functional properties, rice concentrates are close to the FS and EC vectors, soybean concentrates are close to the SC and WSI vectors, and pea concentrates are close to the gelling vector. Finally, in terms of nutritional composition, soybean concentrates are close to the protein vector and pea concentrates to the fat, moisture and starch vectors. These results are in agreement with the results discussed from the data collected in Table 1, Table 2 and Table 3.

Another interesting observation that can be obtained from Figure 2 is the correlation that exists between the variables. The angles between the vectors approximate the correlations between variables in such a way that small acute angles are associated with variables that are strongly positively correlated, obtuse angles close to 180° with variables that are strongly negatively correlated, and right angles with uncorrelated variables [80]. Following this approach, the correlations that can be seen in Figure 2 support the results already discussed from Pearson’s correlation. In this way, it is observed that Mg, K and Ca contents are positively related to WSI, OHC and SC properties and negatively related to FC. Meanwhile, Na and P contents are positively related to WHC and EC and negatively related to ES and FS. In addition, the Cu and Fe contents are positively related to FS and ES properties and negatively related to EC and gelling, while Mn is related to gelling. Considering the acute angles found, the strongest positive correlations occur in the case of Mg/WSI and Na/WHC. As previously stated, the WSI value is related to the soluble solids of the concentrate, which involves not only proteins but also other soluble compounds, like minerals. In this sense, Mg ions have the ability to dissolve rapidly in water [82] which could justify their correlation with this parameter that we found for protein concentrates. In relation to Na, this is the macroelement with the highest concentration in all the concentrates analysed. The relationship between Na and WHC has already been shown previously in flours, where it was observed that an increase in NaCl concentration leads to an increase in WHC [83]. According to these authors, a higher concentration of Na ions seems to increase the electrostatic repulsion of proteins, which would allow more water to bind to the negative charges of proteins, leading to an increase in WHC of flour samples. Similar observations have been described in meat products, where the presence of sodium ions leads to a higher WHC in cooked products, with the effect of Na ions being greater than that of other ions such as K [84].

The techno-functional properties of concentrates are influenced by several factors. On the one hand, they are affected by factors such as variety, origin or amino acid sequence [85]. Thus, some amino acids have shown a high linear correlation with techno-functional properties, although the quaternary structure of the protein seems to have a greater influence than the primary structure or the amino acid profile [86]. Kohnhorst et al. [87] suggested that gelation capacity depends not only on the protein concentration, but also on the non-protein components present in the concentrates. The influence on techno-functional properties of the presence of compounds such as polysaccharides, alkaloids, phenolic compounds or phytates has also been addressed [79]. The polypeptide composition and structure of the proteins in concentrates greatly influence their physicochemical properties and functions [88]. On the other hand, extraction processes also influence these properties, which makes it very difficult to establish functional properties specific to a raw material, depending on the combination of protein raw material and production method [67]. Finally, the methods of determination of the techno-functional properties have a strong influence on the results obtained and therefore, as suggested by Langendörfer et al. [86], the results of the techno-functional properties must always be related to the conditions and method of measurement.

In spite of this, there is hardly any information on the relationship between minerals and these properties in protein concentrates. Based on the results obtained, further studies should be carried out to study these relationships.

## 4. Conclusions

The nutritional composition of the protein concentrates showed differences not only according to the botanical origin of the concentrate but also between different commercial brands, mainly affecting the fat, protein and ash contents. In terms of mineral composition, all the concentrates analysed showed high contents of Na, P, Ca and Mg, with rice concentrates having the lowest mineral concentrations. Differences between commercial brands were observed for all the concentrates analysed. In relation to the techno-functional properties, soybean was highlighted for its higher gelling and swelling capacity and its higher solubility, while rice had a greater capacity for the formation and stability of foams. Differences were observed according to botanical origin except for emulsifying capacity, and differences between commercial brands were observed for all the properties analysed except for water absorption capacity and solubility index. The physicochemical and mineral compositions have been shown to correlate with the techno-functional properties of protein concentrates. Macroelements such as Na and Mg are significantly related to most of the techno-functional properties, while K and Ca are significantly related to SC. As for microelements, Fe and Cu are related to EC and gelling capacity and Zn to SC. Based on the results achieved, the characterisation of the nutritional and mineral composition of protein concentrates may be of great interest in order to anticipate the functional behaviour of these products. Further studies with more varieties of concentrates and commercial brands would be necessary to validate these observed correlations.

## Figures and Tables

**Table 1:** Proximate composition of different protein concentrates (rice, pea, soybean). Data are presented as means ± standard deviation from the triplicate analysis.

	Rice Proteins		Pea Proteins		Soybean Proteins	
	Brand 1	Brand 2	Brand 1	Brand 2	Brand 1	Brand 2
Moisture (%)	4.35 ± 0.08 ^a^	5.43 ± 0.24 ^c^	7.15 ± 0.05 ^d^	7.19 ± 0.04 ^d^	5.71 ± 0.26 ^c^	4.86 ± 0.03 ^b^
Proteins (g/100 g dw)	87.00 ± 0.05 ^b^	87.77 ± 0.23 ^c^	84.35 ± 0.07 ^a^	84.85 ± 0.14 ^a^	89.60 ± 0.39 ^d^	89.80 ± 0.24 ^d^
Fat (g/100 g dw)	4.40 ± 0.01 ^b^	6.35 ± 0.02 ^c^	9.37 ± 0.18 ^e^	8.31 ± 0.13 ^d^	2.65 ±0.01 ^a^	2.63 ± 0.02 ^a^
Carbohydrates (g/100 g dw)	7.36 ± 0.05 ^e^	3.41 ± 0.02 ^d^	2.38 ± 0.02 ^b^	2.41 ± 0.12 ^b^	1.29 ±0.01 ^a^	2.76 ± 0.03 ^c^
Starch (g/100 g dw)	1.72 ± 0.01 ^d^	1.01 ± 0.05 ^a^	1.46 ± 0.10 ^c^	2.06 ± 0.02 ^e^	1.18 ± 0.02 ^b^	0.99 ± 0.01 ^a^
Total sugar (g/100 g dw)	<1	<1	<1	<1	<1	<1
Fibre (g/100 g dw)	<1	<1	<1	<1	<1	<1
Ash (g/100 g dw)	1.27 ± 0.02 ^a^	2.17 ± 0.05 ^b^	3.70 ± 0.22 ^c^	4.29 ± 0.08 ^d^	5.12 ± 0.02 ^e^	4.86 ± 0.02 ^e^
Energy (Kcal/100 g)	417.04 ± 0.09 ^c^	421.86 ± 0.99 ^d^	431.25 ± 1.85 ^e^	423.89 ± 1.40 ^d^	387.46± 1.58 ^a^	393.85 ± 1.11 ^b^

^a–e^: Values followed by different superscripts in the same row are significantly different (*p* < 0.05).

**Table 2:** Mineral composition of different commercial proteins (rice, pea, soybean). Data are presented as means ± standard deviation from the duplicate analysis.

	Rice Proteins		Pea Proteins		Soybean Proteins	
	Brand 1	Brand 2	Brand 1	Brand 2	Brand 1	Brand 2
ppm						
Na	1861.82 ± 31.01 ^b^	816.24 ± 16.36 ^a^	11,530.00 ± 153.61 ^d^	10,719.10 ± 7.88 ^c^	10,944.44 ± 67.13 ^c^	13,307.24 ± 10.41 ^e^
Mg	330.50 ± 1.47 ^b^	179.52 ± 2.71 ^a^	707.86 ± 7.79 ^d^	429.35 ± 16.69 ^c^	971.99 ± 11.79 ^f^	799.06 ± 0.76 ^e^
P	2650.93 ± 2.94 ^a^	3270.61 ± 26.44 ^b^	8083.31 ± 106.50 ^d^	7611.91 ± 17.50 ^c^	7730.74 ± 30.45 ^c^	8176.00 ± 26.03 ^d^
K	78.65 ± 1.36 ^b^	42.20 ± 0.01 ^a^	1224.59 ± 0.65 ^d^	447.74 ± 4.93 ^c^	2027.68 ± 1.38 ^e^	2410.37 ± 4.19 ^f^
Ca	688.41 ± 4.37 ^a^	1043.30 ± 20.86 ^b^	1421.15 ± 11.52 ^c^	1423.29 ± 14.57 ^c^	5225.22 ± 11.55 ^e^	4418.93 ± 22.91 ^d^
Mn	25.51 ± 0.11 ^d^	31.48 ± 0.27 ^e^	10.98 ± 0.15 ^b^	5.84 ± 0.04 ^a^	13.15 ± 0.47 ^c^	13.81 ± 0.16 ^c^
Fe	180.00 ± 0.35 ^c^	87.08 ± 1.30 ^a^	256.15 ± 3.46 ^d^	267.77 ± 3.74 ^e^	132.34 ± 2.81 ^b^	133.14 ± 0.94 ^b^
Cu	14.64 ± 2.23 ^b,c^	17.69 ± 0.24 ^c^	11.18 ± 0.20 ^a,b^	8.95 ± 0.00 ^a^	12.00 ± 0.00 ^a,b^	11.59 ± 0.16 ^a,b^
Zn	59.49 ± 0.21 ^b^	73.95 ± 0.70 ^e^	67.77 ± 0.95 ^d^	63.07 ± 0.11 ^c^	20.42 ± 0.04 ^a^	21.75 ± 0.25 ^a^
ppb						
Se	496.21 ± 22.08 ^b,c^	613.30 ± 45.04 ^c^	436.15 ± 2.53 ^b^	1039.83 ± 35.33 ^d^	200.95 ± 60.32 ^a^	185.51 ± 23.46 ^a^
Ni	240.22 ± 19.88 ^a^	604.54 ± 65.39 ^b^	585.49 ± 47.94 ^b^	268.27 ± 35.21 ^a^	1447.33 ± 35.89 ^c^	2282.92 ± 30.11 ^d^
Cr	208.98 ± 4.31 ^a^	627.11 ± 2.34 ^b^	78.97 ± 11.24 ^a^	114.11 ± 31.83 ^a^	103.74 ± 12.28 ^a^	189.71 ± 88.97 ^a^
Cd *	nd	nd	nd	nd	nd	nd
Pb **	nd	175.40 ± 3.15	nd	nd	nd	nd

^a–f^: Values followed by different superscripts in the same row are significantly different (*p* < 0.05). * Detection limit < 0.015. ** Detection limit < 0.026.

**Table 3:** Techno-functional properties of commercial proteins. Data are presented as means ± standard deviation.

	Rice Proteins		Pea Proteins		Soybean Proteins	
	Brand 1	Brand 2	Brand 1	Brand 2	Brand 1	Brand 2
WHC (g/g)	3.46 ± 0.06 ^b^	2.13 ± 0.03 ^a^	5.18 ± 0.13 ^d^	4.56 ± 0.32 ^c^	5.94 ± 0.27 ^e^	4.84 ± 0.26 ^c,d^
OHC (g/g)	0.99 ± 0.09 ^a^	1.00 ± 0.11 ^a^	1.16 ± 0.03 ^b^	1.12 ± 0.09 ^a,b^	1.08 ± 0.05 ^a,b^	1.29 ± 0.03 ^c^
WSI (°Brix)	1.0 ± 0.00 ^a^	1.7 ± 0.06 ^a^	11.7 ± 0.06 ^b^	11.0 ± 0.10 ^b^	18.3 ± 0.06 ^c^	22.0 ± 0.10 ^d^
SC (%)	0.74 ± 0.01 ^b^	0.25 ± 0.07 ^a^	1.35 ± 0.04 ^d^	1.05 ± 0.03 ^c^	2.43 ± 0.08 ^f^	2.08 ± 0.06 ^e^
FC (%)	474.60 ± 2.75 ^e^	294.66 ± 8.39 ^d^	257.57 ± 10.50 ^c^	296.82 ± 5.50 ^d^	228.61 ± 3.37 ^b^	136.36 ± 31.8 ^a^
FS (%)	465.08 ± 2.75 ^d^	284.33 ± 17.21 ^c^	87.88 ± 5.25 ^b^	49.20 ± 5.50 ^a^	101.39 ± 2.41 ^b^	92.42 ± 2.62 ^b^
EC (%)	43.54 ± 2.12 ^b^	35.62 ± 0.74 ^a^	41.61 ± 2.06 ^a,b^	63.25 ± 3.84 ^c^	59.88 ± 1.45 ^c^	45.59 ± 1.90 ^b^
ES (%)	82.54 ± 1.15 ^e^	77.13 ± 0.65 ^d^	75.05 ± 2.57 ^d^	55.19 ± 2.77 ^b^	48.75 ± 0.86 ^a^	67.83 ± 0.37 ^c^
GF (N)	85.39 ± 24.66 ^b^	59.79 ± 1.14 ^a^	639.90 ± 1015.93 ^c^	619.08 ± 158.51 ^c^	2067.33 ± 1870.70 ^d^	2482.80 ± 218.24 ^d^
a_w_	0.344 ± 0.001 ^c^	0.318 ± 0.004 ^b^	0.404 ± 0.001 ^f^	0.389 ± 0.001 ^e^	0.371 ± 0.002 ^d^	0.134 ± 0.002 ^a^
L*	82.03 ± 27.38 ^d^	78.08 ± 1.17 ^c^	76.36 ± 2.42 ^b,c^	73.52 ± 1.83 ^a^	74.69 ± 2.14 ^a,b^	83.53 ± 2.14 ^d^
a*	2.71 ± 0.07 ^d^	2.46 ± 0.07 ^c^	5.06 ± 0.01 ^e^	5.39 ± 0.01 ^f^	0.37 ± 0.07 ^a^	0.51 ± 0.02 ^b^
b*	15.91 ± 0.16 ^a^	19.16 ± 0.35 ^b^	25.00 ± 0.61 ^e^	23.23 ± 0.47 ^d^	19.96 ± 0.48 ^c^	18.77 ± 0.37 ^b^

WHC: water-holding capacity; OHC: oil-holding capacity SC: swelling capacity; WSI: water solubility index; FC: foaming capacity; FS: foaming stability; GF: gel formation (hardness of the gel formed at pH = 5); EC: emulsion activity; ES: emulsion stability; L*: lightness; a*: redness; b*: yellowness. ^a–f^: Values in the same row followed by different letters are significantly different (*p* < 0.05).

## Data Availability

The original contributions presented in this study are included in the article. Further inquiries can be directed to the corresponding author.
